# Editorial: The Molecular Basis of Somatic Evolution

**DOI:** 10.3389/fonc.2022.906068

**Published:** 2022-06-06

**Authors:** Qiyuan Li, Bing Xu, Zhanlong Shen

**Affiliations:** ^1^ Department of Hematology, The First Affiliated Hospital of Xiamen University and Institute of Hematology, School of Medicine, Xiamen University, Xiamen, China; ^2^ National Institute for Data Science in Health and Medicine, Xiamen University, Xiamen, China; ^3^ School of Medicine, Xiamen University, Xiamen, China; ^4^ Department of Hematology, Key Laboratory of Xiamen for Diagnosis and Treatment of Hematological Malignancy, Xiamen, China; ^5^ Laboratory of Surgical Oncology, Peking University People’s Hospital, Beijing, China; ^6^ Laboratory of Surgical Oncology, Beijing Key Laboratory of Colorectal Cancer Diagnosis and Treatment Research, Beijing, China

**Keywords:** cancer, somatic evolution, mutational signature, intratumor heterogeneity (ITH), driver gene

Cancer cells acquire malignant phenotypes by various somatic alterations in the genome (1). The acquisition of these phenotypes is the result of the selection pressures to evolve highly proliferative, metastatic, and resistant clones. The processes of both intrinsic and extrinsic selection drive the somatic evolution, shape the landscape of the cancer genome, and directly determine the clinical risks and benefits to the patients (2). Signatures of biological processes which alter the mutational patterns of cancer genomes are found and annotated in many cancers (3). Such signatures can be used to trace the formation of clonality during different stages of somatic evolution and consequential intra- and inter-tumor heterogeneity (4, 5).

Nevertheless, the mutational signatures alone cannot fully reveal the complex biological processes underlying somatic evolution. The internal and external determinants of somatic evolution, with the relevant mechanisms are still largely unknown. In addition, other selection processes, driven by new, highly specific determinants are yet to be discovered (6).

The answers to these questions are keys to the precision medicine of cancers. For many years, researchers have determined relevant mutations as “cancer drivers” based on the sheer frequency of mutation among patients. This strategy has yielded many successful clinical applications (7). The logic behind this is that the most frequent mutations are surrogates for the most prevalent clones in the somatic evolution of cancer, many of which determine the fitness of cancer in somatic selection (8).

To obtain more details of the process of somatic evolution at the mechanistic level is one of the most appealing but challenging tasks in oncology. It is also a field where new high-throughput technologies and multi-omics analyses play more and more active roles to uncover the molecular basis of the somatic evolution (6, 9).

There are both intrinsic and extrinsic determinants of somatic evolution. At the intrinsic level, germline cancer risk loci act as determinants of fitness in the early stage of tumorigenesis. For example, BRCA1 deficiency is likely to inflict diverse forms of genomic lesions (collapsed forks or double-strand breaks) which cause multiple signatures including homologous recombination deficiency (HRD) signatures and insertion or deletion (ID) signatures (10). Germline MC1R status is associated with somatic C>T mutations, a signature linked to sun exposure in melanoma (11). Somatic mutations are another major type of intrinsic determinants of cancer; many are known as cancer driver genes. Somatic mutations in tumor suppressors are crucial in tumorigenesis according to the “two-hit” hypothesis. Somatic driver mutations also determine the clonality and intratumor heterogeneity (ITH) in many cancer types (12, 13). Many of these somatic mutations have been proved to alter the fitness of cancer cells *in vitro* (14) and *in vivo*, hence somatic selection. In particular, somatic structural variations (SVs) are more functionally active in somatic evolution. Scott A Tomlins et al. demonstrated, in a FISH-based study, that ERG-TMPRSS2 might be the key lesion driving the invasive transition but not transformation from PIN lesions in prostate cancer (15). Hu et al. report a case of a pediatric central nervous system embryonal tumor with CIC–LEUTX fusion, which is closely associated with oncogenic alteration because of its chimeric transcriptional regulatory properties. Wang et al. report a case of follicular lymphoma (FL) with novel CIITA_CREBBP fusion and CREBBP mutations, which is associated with the functional loss of CREBBP. The case shows a high Ki-67 proliferation index in the low-grade FL.

Yet many more somatic selection processes of cancer cells are extrinsic. Extrinsic selection includes a wide spectrum of complex biological mechanisms including environmental, therapeutic, and immune microenvironment processes (16). Exposure to environmental mutagens is known to increase cancer risk. Dai et al. showed that long-term low-dose cadmium (Cd) from cigarette smoking introduced a series of somatic alterations in the genome of A549 cells, together with other transcriptomic and morphological changes which are associated with malignant phenotypes. Further analysis suggested that Cd exposure influences cellular activities in cell adhesion, movement, and metabolism. The study provides an unusual longitudinal view of somatic evolutionary trajectories under environmental exposure.

Metastatic tumors represent a unique stage in somatic evolution where the new clones are capable of migrating to other organs. Chung et al. screened 1,120 compounds for cytotoxic effects in different metastases in prostate cancer and showed a subset of compounds including fenbendazole, fluspirilene, clofazimine, niclosamide, and suloctidil with selective efficacy in metastatic prostate cancer cells *in vitro* and *in vivo*.

Piece by piece, findings of the molecular basis of somatic evolution will facilitate the identification of new cancer biomarkers and targets for therapy, which will further improve the clinical management of cancer.

## Author Contributions

QL, BX, and ZS contributed to the conception and design of the study. QL wrote the first draft of the manuscript. BX and ZS were responsible for the revision of the manuscript for important intellectual content. All authors contributed to manuscript revision, read, and approved the submitted version.

## Conflict of Interest

The authors declare that the research was conducted in the absence of any commercial or financial relationships that could be construed as a potential conflict of interest.

## Publisher’s Note

All claims expressed in this article are solely those of the authors and do not necessarily represent those of their affiliated organizations, or those of the publisher, the editors and the reviewers. Any product that may be evaluated in this article, or claim that may be made by its manufacturer, is not guaranteed or endorsed by the publisher.
